# Developing a Heatwave Early Warning System for Sweden: Evaluating Sensitivity of Different Epidemiological Modelling Approaches to Forecast Temperatures

**DOI:** 10.3390/ijerph120100254

**Published:** 2014-12-23

**Authors:** Christofer Åström, Kristie L. Ebi, Joakim Langner, Bertil Forsberg

**Affiliations:** 1 Public Health and Clinical Medicine, Occupational and Environmental Medicin, Umeå University, SE901 87 Umeå, Sweden; E-Mails: Ekrisebi@essllc.org (K.L.E.); bertil.forsberg@envmed.umu.se (B.F.); 2 Swedish Meteorological and Hydrological Institute, SE601 76 Norrköping, Sweden; E-Mail: joakim.langner@smhi.se

**Keywords:** heatwave, early warning, forecast

## Abstract

Over the last two decades a number of heatwaves have brought the need for heatwave early warning systems (HEWS) to the attention of many European governments. The HEWS in Europe are operating under the assumption that there is a high correlation between observed and forecasted temperatures. We investigated the sensitivity of different temperature mortality relationships when using forecast temperatures. We modelled mortality in Stockholm using observed temperatures and made predictions using forecast temperatures from the European Centre for Medium-range Weather Forecasts to assess the sensitivity. We found that the forecast will alter the expected future risk differently for different temperature mortality relationships. The more complex models seemed more sensitive to inaccurate forecasts. Despite the difference between models, there was a high agreement between models when identifying risk-days. We find that considerations of the accuracy in temperature forecasts should be part of the design of a HEWS. Currently operating HEWS do evaluate their predictive performance; this information should also be part of the evaluation of the epidemiological models that are the foundation in the HEWS. The most accurate description of the relationship between high temperature and mortality might not be the most suitable or practical when incorporated into a HEWS.

## 1. Introduction

Over the last two decades, a number of heat extremes resulted in devastating health outcomes around the globe. The 1995 heatwave in Chicago resulted in health consequences far beyond previous experiences, with at least 700 excess deaths from July 14 through July 17 [1]. In Europe, the heatwave of 2003 dramatically demonstrated to the public that heatwaves could pose a significant public health threat. In France alone, more than 14,000 people died [2]. In Moscow in 2010, a heatwave combined with widespread forest fires resulted in more than 11,000 excess deaths [3]. 

Recent events underscore the need for heatwave early warning systems (HEWS), bringing the need for such systems to the attention of many European governments. Public health institutes and weather services around Europe have mainly been responsible for the design of these warning systems. The approach for designing warning systems differs across countries, including different exposure variables and thresholds predicting the temperature at which mortality increases markedly, and different interventions from country to country [4]. 

There is not a consensus on which exposure variable is the best predictor of heat-related mortality and morbidity. Few studies have investigated which temperature variable performs best in modelling heat-related illness [5,6,7]. One study concluded that the choice of temperature exposure variable can be based on practical concerns [7]. A similar study found that the best index for thermal comfort was not necessarily the best predictor of heat related mortality [8]. 

Different thresholds across countries would be expected based on past experience with high ambient temperatures, the extent to which infrastructure is designed to protect occupants from higher temperatures, and other factors. In fact for some countries, thresholds differ between different national regions due to different climates. 

The temperature thresholds used in a HEWS are generally based on epidemiological studies that investigated historical temperature observations and mortality. These thresholds are then operationalized in the system under the apparent assumption there is a high correlation between forecast and observed exposure variables, but for HEWS it is the forecasts of extreme temperatures that is critical.

The aim of this study is to investigate the use of forecasted temperatures in addition to observations when evaluating the underlying epidemiological models within a heatwave early warning system. We first investigated the accuracy of temperature forecasts by comparing them with observations. We next evaluated the sensitivity of different statistical models and exposures of the temperature mortality relationship when using forecasted *vs.* observed temperatures. Finally, we investigated how these sensitivities limit how far in advance of an impending heatwave an accurate forecast could be issued.

## 2. Experimental Section 

### 2.1. Data

Mortality and weather data were collected for the time-period 1998–2007. We used daily data on non-accidental mortality for Stockholm County, collected from the Cause of Death Register at the Swedish Board of Health and Welfare. The Swedish Meteorological and Hydrological Institute (SMHI) provided temperature data measured at Observatorielunden, an urban weather station in central Stockholm. SMHI also provided daily forecasts of 2-meter temperature from the European Centre for Medium-range Weather forecasts, ECMWF, for the summer months, April to September 1998–2007, including forecasts one, two and three days in advance. The temperature forecasts were direct model output extracted from the 00 UTC daily operational, deterministic ECMWF forecast for the location of the Observatorielunden station in Stockholm. Daily maximum temperatures were estimated as the maximum temperature in the forecast based on three or six-hourly data depending on time period and forecast length. It should be noted that the forecast data represent spatial averages over the computational grid used in the ECMWF forecast model which was ca. 50 × 50 km^2^ in the period 1998–2005 and 25 × 25 km^2^ in 2006–2007. Higher resolution forecast models have been in use in Sweden for part of the time period studied. The ECMWF data was chosen because of consistency for the time period studied. Also for the longer forecast lead times (beyond 2 days) ECMWF was the best source of information for the studied time period.

### 2.2. Study Design

We compared temperature forecasts with the observed temperatures to identify any patterns and consistent differences. The observed temperatures and the forecast data were compared by modelling the observed temperature with the forecast temperatures as explanatory variables. If any systematic bias was present, an adjusted forecast temperature might increase model accuracy.

In the second stage, we studied four statistical approaches to model temperature mortality relationships. The base model was constructed as: Mortality~Poisson(*µ_t_*):

$$Log\left(\mu_{t}\right)=intercept+\;wd_{t}+S\left(trend_{t}\right)+f\left(temperature\right)$$

where *wd_t_* it the day of the week, *S*(*trend_t_*) is a smooth function for the time trend over the entire period and a function of temperature depending on the underlying statistical model.

We used different approaches commonly used in similar studies to model the relationship between temperature and total mortality in the population [9,10,11,12]. Four models were chosen with different levels of complexity: distributed lag non-linear models (DLNM) [13]; a generalized additive model (GAM) [14] with a smooth spline function; a piecewise linear (PWL) relationship; and models incorporating temperature as a threshold effect (THR). As the aim of this study was to investigate the sensitivity of a underlying model when used with weather forecasts instead of observations, we used experience from existing studies on temperature related mortality in the Stockholm area rather than make a thorough investigation of our own. We used a lag 0–2 mean of daily maximum temperature in the models; this is the lag used in the Swedish HEWS being testing and is based on experiences from warning systems in other European countries where 3-day average temperatures are commonly used [4]. 

Finally, we investigated whether models using temperature forecasts would identify days considered risk-days and whether there are any differences among the four modelling approaches. The cut-off for what was considered a risk day was decided to be a level of risk increase. We used the estimated risk at 26 °C in the PWL model. The temperature was taken from the lower level in the Swedish HEWS. The PWL model was chosen because it is the least complex model with a continuous exposure space. We also compared the estimated risk increases produced by the observed and forecast temperatures to identify any patterns suggesting a bias that could be corrected when using forecast temperatures to issue a warning. This also helps identify how far in advance an accurate warning could be issued. The models were evaluated using sensitivity and positive predictions value (PPV). Sensitivity is a measure of how well the model identifies the actual risk days. PPV describes how large a proportion of the risk days classified by the model were actual risk days. This means that a high PPV would result in few false positive forecasts. Both measures range from 0 to 1 where 1 is perfect classification. Specificity, which describes the ability to identify non-risk days, is not shown because almost 95% of the days were non-risk days, leading to very high specificity scores for all models. 

## 3. Results and Discussion

### 3.1. Forecasts

The forecast temperatures showed a clear bias, especially in the warm end of the distribution with the forecasts generally estimating lower temperatures than were observed. A linear function can be used to adjust forecast temperatures to reduce this bias over the period 1998–2007 (Table 1).

**Table 1:** Estimated coefficients when observed temperatures are used to model forecast temperatures.

Forecast Length	Intercept	Slope
1-day forecast	2.283	0.829
2-day forecast	2.388	0.825
3-day forecast	2.753	0.795

**Figure 1:**
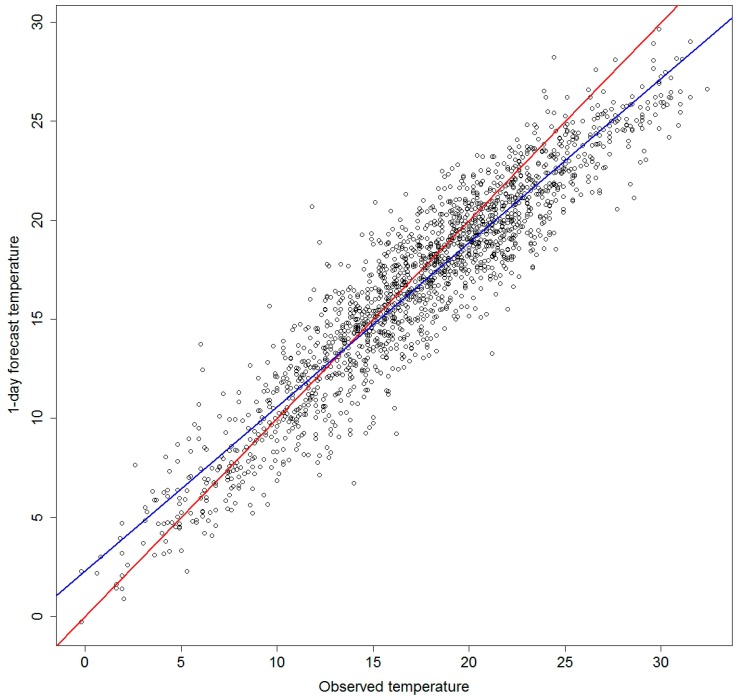
Observed *vs.* 1-day forecast temperatures, Stockholm, 1998–2007. Red line shows the linear estimate for an unbiased fit and the blue the estimated linear relationship between the variables.

The increasing intercept and decreasing slope for longer time frames indicate that the forecast bias is not only present for all time frames, but also will increase with longer forecasts. These results made us investigate how the models perform using adjusted and un-adjusted forecast temperatures. We calculate the adjusted temperatures using the linear models from Table 1. But even after correction, the forecast temperatures were still generally lower in the high end of the distribution (Figure 1).

### 3.2. Risk Models

In all four models, we modelled the long-term trend as a penalized thin plate regression spline with maximum 12 degrees of freedom (d.f.) for each year. Of the possible 120 d.f., the penalized spline used between 69.7 and 72.33 effective d.f. for the different models. This smooth function accounts for annual patterns and the long-term time trend.

The thresholds used in the THR model are the same as in the underlying model in the Swedish HEWS. This modelling approach incorporated temperature as a three level ordinal factor variable. The temperature limits used were 20, 27 and 30 °C. A 26 and 30 °C threshold being tested in the Swedish HEWS, where the lower limit was set lower since warnings are issued when the threshold is expected to be exceeded for an area of 1000 km^2^. At these temperatures the threshold model found statistically significant increases in the daily number of deaths of 3.1, 10 and 20.5%. These estimated increases correspond to the temperature ranges 20–27 °C, 27–30 °C and above 30 °C. The reason to only include the two higher threshold values in the Swedish HEWS is explained by the low risk increase estimated for the lowest threshold. Overall, this model explains 17.6% of the variation in daily mortality.

The PWL model to describe the association uses the 90th percentile of the 0–2 lag maximum temperature, 21.7 °C, as the break point. The model estimates a risk increase of 1.4% per degree increase above the 90th percentile and explains 17.6% of the variation. This corresponds to risk increases of 7.5 and 11.9% at 27 and 30 °C, respectively. To be able to compare estimates to the threshold model we calculated the mean of risk estimates for the temperature ranges 27–30 °C and above 30 °C. These were estimated to 9.4% and 12.7%.

When modelling the temperature effect in the GAM model, we used a penalized smooth spline with 8 d.f.. There was a significant risk increase estimated to be 7.0 and 10.4% at the 27 °C and 30 °C thresholds, respectively. The mean of the estimated risk increase for the observations in the 27–30 °C interval is 8.8% and 11.1% above 30 °C. This model explains 17.7% of the variation in temperature-mortality relationships.

The DLNM model incorporated temperature as a function using 5 d.f. for temperature and 3 d.f. for the lag. The time lag was set to 20 days. This model produced the lowest risk estimates in the lower interval, with a mean risk increase of 4.3%. In the above 30 °C interval, the DLNM produced a mean risk increase of 13%. It should be noted that the DLMN model results were based on lag 0–2 to be comparable to the other models. The model explains 18.1% of the variation.

The large difference in the estimated risk increase in the above 30 °C interval made it necessary to conduct a sensitivity analysis to investigate the effects of temperatures above 30 °C. We added an indicator variable for days with 0–2 lag max temperatures above 30 °C in the PWL and the GAM models. In these models, these indicator variables are estimated to be 8.9 and 11.1%, respectively (*p*-values of 0.179 and 0.093). Additionally we investigated whether any of the observations in the highest interval was very influential for the estimates using a leave one out routine. We estimated the risk increase in the highest temperature interval using the THR model with one of the observations removed. This was repeated for all observations in the highest temperature interval. If any observations was too influential in this interval the risk estimate produced when this observation was removed would be significantly lower than the rest. All estimates were significant and the effect estimate ranged from 17.4% to 24.2% depending on which observation was removed.

**Figure 2:**
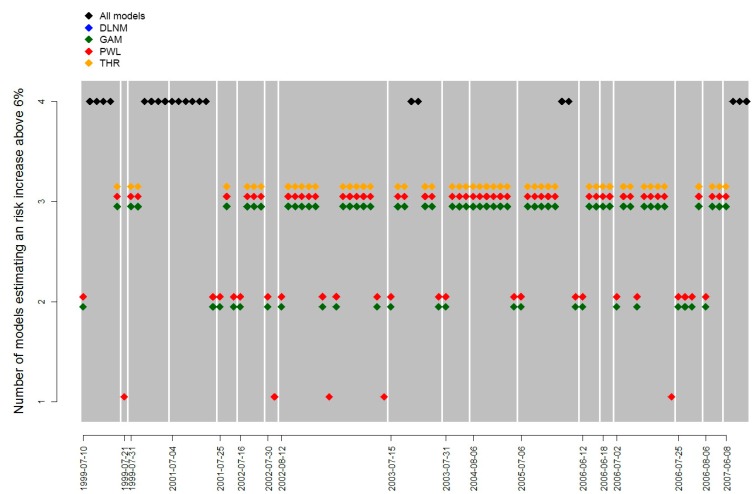
Comparison of which models identify heatwave days as risk days. Each grey area represents a period where risk days were identified. The markers describe which models classified each day as a risk day. The date on the x-axis describes the first day of each period with elevated mortality risk.

To assess the predictive ability of the models we looked at whether the models were able to identify risk days. The risk increase used as a cut off value was estimated to be 6% which corresponds to the estimated risk increase at 26°C for the PWL model. We estimated the elevated risk due to temperatures for all days with each of the models. All days identified as a risk day, with risk estimates above 6%, by any of the models were compiled in a separate dataset. We investigated which models identified these days as risk days to find the level of agreement between models (Figure 2). 

Figure 2 shows that across the models, there were 98 days identified as a risk day. Among these, there were 70 days where three or more models agreed it was a risk day. As expected, the PWL model identified the most risk days as it was used to define what constitutes a risk day. The GAM model shows very high agreement with the PWL model. The DLNM model classifies the fewest days as risk days.

For each model we calculated estimated risk for observed as well as forecast temperatures, using both adjusted and unadjusted forecasts. The estimates were compared between observed and forecasts for each model separately. The sensitivity and positive prediction values (PPV) are presented in Table 2 and Table 3.

**Table 2:** Sensitivity scores for the four models using forecast and adjusted forecast temperatures, and different issuing time frames.

Model Type	Forecast			Adjusted Forecast		
	1-day	2-day	3-day	1-day	2-day	3-day
DLNM	0.62	0.05	NA	0.67	0.10	NA
GAM	0.78	0.53	0.16	0.86	0.72	0.41
PWL	0.79	0.56	0.19	0.88	0.72	0.47
THR	0.70	0.30	0.09	0.84	0.57	0.23

**Table 3:** Positive prediction values for the four models using forecast and adjust forecasted temperatures, and different issuing time frames.

Model Type	Forecast			Adjusted Forecast		
	1-day	2-day	3-day	1-day	2-day	3-day
DLNM	1.00	1.00	NA	0.88	0.50	NA
GAM	1.00	0.98	0.94	0.99	0.94	0.93
PWL	1.00	1.00	1.00	0.98	0.97	0.93
THR	0.96	0.95	1.00	0.97	0.91	0.89

The DLNM was the model most sensitive to forecast temperatures. All models improve when used with the adjusted forecast temperatures, especially for longer time frames. But even though the performance increases, the accuracy is very low when looking at three-day temperature forecasts for all models. The PWL model does however seem the most robust when used with forecasts. 

**Figure 3:**
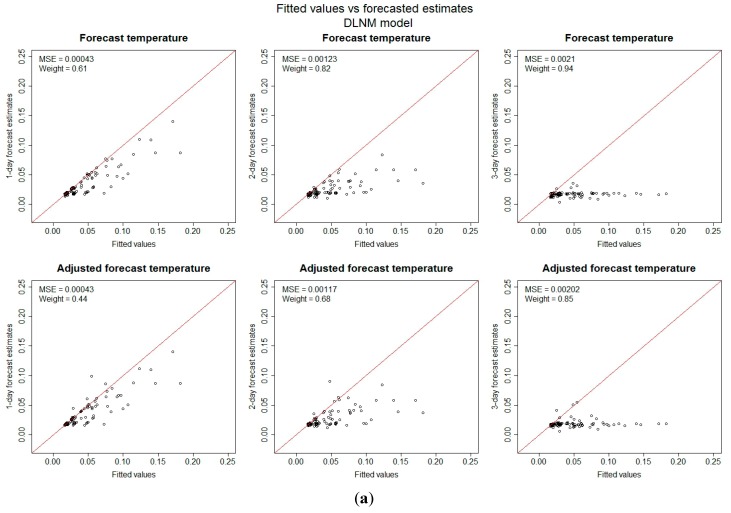

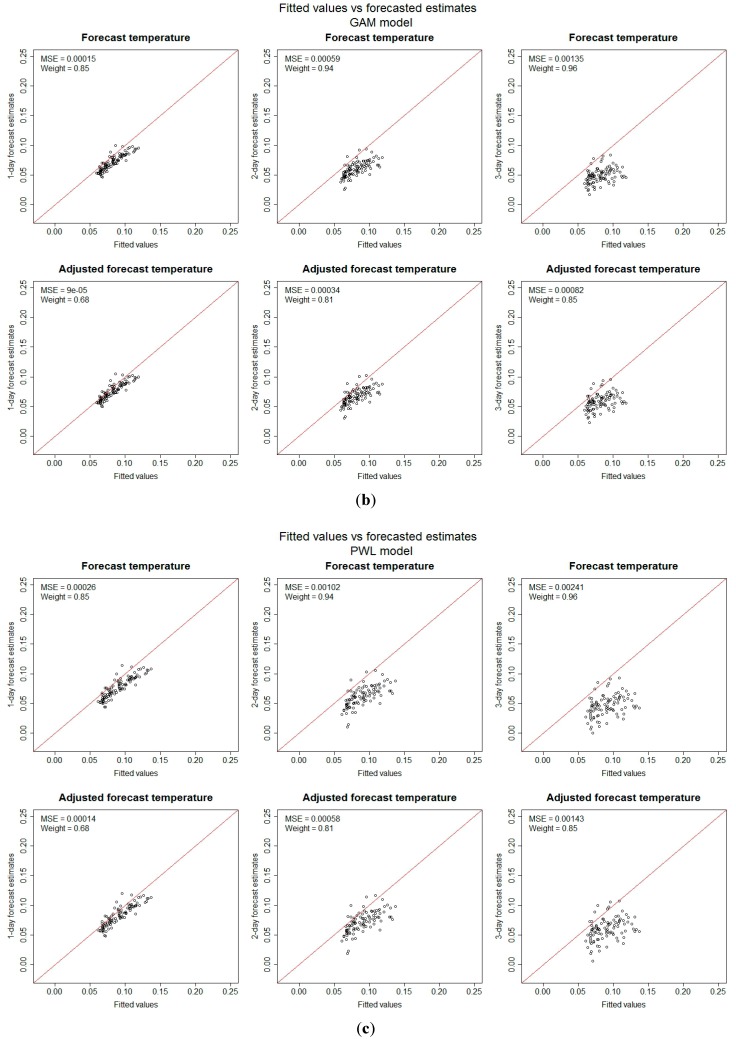

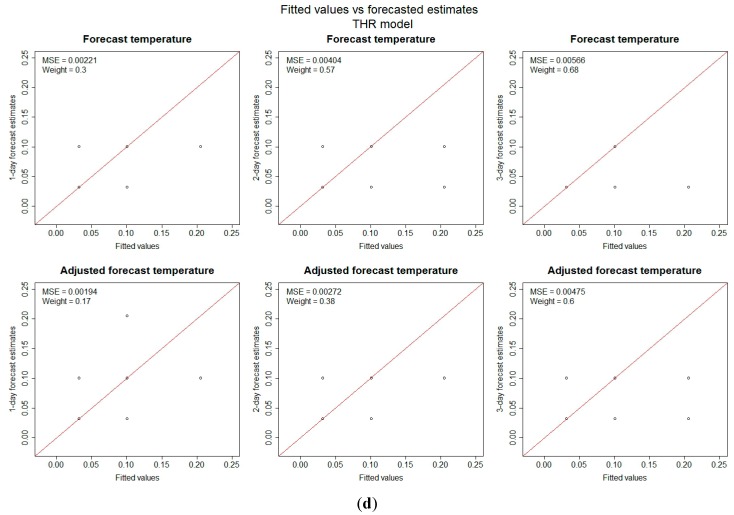
Risk estimates for the four models using fitted values from observed temperatures and the forecast temperatures on days with temperature above 26 °C. On the x-axis is the estimated risk increases in percent produced by observed temperatures and on the y-axis the estimated risk increase produced by different forecast times for each day in the study period for the: (**a**) DLNM model; (**b**) GAM model; (**c**) PWL model; (**d**) THR model.

When looking at the actual risk estimates using forecasts and not the dichotomous variable risk or non-risk day, the differences between the models are clearer. The models were evaluated by the mean square error (MSE) and the weight (Figure 3). 

The weight is a variable between −1 and 1 that measures how large a portion of the estimates from one model is higher than the other. For any given day, if the estimates using observed temperature are higher than the estimates using forecast temperatures for that day, it is assigned a 1 and -1 otherwise. The weight is a mean value of this indicator for all days in the period. A value of 1 would mean that the all estimates using observed temperature were higher than the ones using forecast temperatures and −1 the opposite. A value of 0 would indicate a balance between the two models but nothing about the accuracy. 

The DLNM model underestimated the risks when the forecast length increased beyond 2 days. The GAM and PWL models show slightly better ability when used with adjusted forecast temperatures. The DLNM model has no clear pattern of prediction bias, while there appears to be a pattern forecast bias (under predictions) for the GAM and PWL models.

The risk estimates in the THR model are hard to compare with the models with a continuous outcome. The sensitivity and PPV however show that it has a high ability to classify risk days at least over shorter time frames.

## 4. Discussion 

This study shows a discrepancy between estimated risk increases based on direct forecast model output and actual observed temperatures in Stockholm. This discrepancy appears to increase as the forecast time lengthens. This raises the question of whether operating HEWS are adjusting for this forecast bias, and, if so, how. This phenomenon is not only present in Stockholm; another study found similar results [15]. A review of the European HEWS found that several systems have lead times of 3 days or more. In the light of our results and those from other studies, assuming a similar forecast bias exists, then these systems may not be able to issue accurate warnings that far in advance. The practice of providing daily revisions of the forecasts is clearly important. The Swedish and some other HEWS issue warnings for the lower levels based on forecasts alone and combine observed and forecast temperatures to issue more accurate warnings for the higher levels. It should be noted that the forecast data used in this study are a proxy for actual forecasts of daily maximum temperatures issued by a weather service. In practice new model forecasts are available more than once a day and different bias corrections are applied. However, such data were not easily available from SMHI for the study period. 

It has recently been proposed to use improved gridded weather data rather than data from a single weather station for studies of heat-mortality relations [16]. Such data should be explored and if interpolated temperature data prove to give more valid associations, they should be included in future evaluations of models for HEWS.

The different models yield slightly different results but all find an increase in mortality with high temperatures. The models mostly identify the same days as risk days, with the exception of the more conservative DLNM model. The fact that 70 out of 98 days were classified as risk days by at least three of the models shows a high level of agreement. However, a model that would miss 28 days of higher than average mortality indicates improvements in heatwave early warning systems could save more lives.

There are discrepancies in risk estimates from the different models. The DLNM is the most conservative, and the THR model estimates the highest risks. The GAM and the PWL models yield similar results. The models using the 0–2 lag of daily maximum temperature produce similar risk increase estimates for the range 27–30 °C (8.8, 9.4 and 10%). In the interval above 30 °C, the difference is much larger between models (11.9, 11.1 and 20.5%). The low number of observations above 30 °C could be an explanation. In the threshold model, the estimated increase in mortality is based on observations for each interval separately. Few observations in an interval would make it sensitive to influential observations. However, our sensitivity analysis indicates that no such influential observations would alter the effects to such extent as shown in the model comparison. The risk estimates from the other models calculate the effects on mortality based on observations from a wider temperature interval, making it conservative in the tails of the exposure distribution. While this is normally a positive attribute, in this case, such conservativeness means missing days when lives could be saved.

Despite the *p*-values of the sensitivity analysis suggesting the 30 °C additional indicator is not significant, the main effects of temperature were slightly lower in terms of risk increase per degree when this indicator was added. If the indicator variable was treated as a significant contributor, the combined effect with the main temperature effect would generate risk estimates similar to the threshold model for temperatures above 30 °C. Although the indicator variable is not significant, the quite low *p*-values suggest there may be effects in the extremes of the distribution that the PWL and GAM models can’t explain. Our suspicions that there was one very influential observation in the highest temperature interval was proved false as all estimated returned significant in the leave one out routine and the estimates was not lower in any case.

The difference between models when using forecast temperatures can be traced back to the nature of the exposure variable and the dose-response relationship. In the case of the DLNM model, the fact that it is based on temperatures on a daily level rather than a 0–2 lag mean temperature makes it more sensitive to inaccurate forecasts. This would be true for all models incorporating the temperature effects on specific lags. The differences between the models using the same exposure variable depend on the steepness of the dose-response curve. A steeper curve in the GAM or PWL model would make it more sensitive as a small change in exposure would result in a large change in risk. 

When examining the forecast risks, the DLNM model error shows no clear bias. The GAM and PWL models however show a clear bias for all time scales that makes it possible to adjust the intercept of the curve to get a significantly better MSE. This reinforces that these models are preferable when compared to models using risk estimates based on temperatures on individual days, which are more sensitive to inaccurate forecasts. However, an adjustment of the intercept could increase the probability of false positives. In the context of a warnings system, one must weigh the accuracy from a statistical point of view with the purpose of the forecast. Too many false positives undermine the warning system and too many false negatives may have substantial health consequences. Finding the right balance between sensitivity and PPV requires consideration of science and the needs and perspectives of the individuals to whom the warning is directed. 

The accuracy of warnings using the forecast data declines when using longer forecasts. This is to be expected. All models have problems forecasting extremes, but this study shows that some exposure variables are more robust than others. Models using only the maximum temperature as exposure, a variable that is fairly easy to forecast compared to variables such as relative humidity, may be less sensitive to forecasts and have higher skill. Models using more complex exposure measures might be less reliable when used to forecast risks in such situations. 

Although complex models can be useful in establishing exposure-response relationships, they may not be suitable when operating a HEWS. This results together with the conclusions that the exposure variable can be chosen based on practical concerns [7], indicate the use of less complex exposure variables and models to decide the levels within a heatwave early warning system benefit not only from being easier to understand by the public but might also have a higher predictive performance.

## 5. Conclusions

Considerations of the accuracy in temperature forecasts should be part of the design of a HEWS. Currently operating systems can to some extent evaluate their predictive performance; this information should also be part of the evaluation of the epidemiological models that are the foundation in the HEWS. The most accurate description of the relationship between high ambient temperature and mortality might not be the most suitable or practical when incorporated into a HEWS. A robust statistical model and temperature exposure variable are important to have accurate forecasts of the expected increase in mortality during a heatwave. 
